# Roles of fasting and postprandial blood glucose in the effect of type 2 diabetes on central arterial stiffness: a 5-year prospective community-based analysis

**DOI:** 10.1186/s13098-017-0231-3

**Published:** 2017-05-12

**Authors:** Shihui Fu, Wenji Chen, Leiming Luo, Ping Ye

**Affiliations:** 1 Department of Geriatric Cardiology, Chinese People’s Liberation Army General Hospital, Beijing, 100853 China; 2 Department of Cardiology and Hainan Branch, Chinese People’s Liberation Army General Hospital, Beijing, China; 3 Department of Rheumatology and Hainan Branch, Chinese People’s Liberation Army General Hospital, Beijing, China

**Keywords:** Central arterial stiffness, Fasting blood glucose, Postprandial blood glucose, Type 2 diabetes

## Abstract

**Background:**

Cardiovascular disease constitutes a major challenge for the health of community-dwelling population, it is essential to delay the development of atherosclerosis. However, long-term prospective studies analyzing the effect of type 2 diabetes (T2D) on central arterial stiffness are lacking, and roles of fasting and postprandial blood glucose (FBG and PBG) in this effect are controversial. Purpose of the current analysis was to investigate the effect of T2D on central arterial stiffness during the 5 years of follow-up, and explore whether both FBG and PBG were determinants of this effect in Chinese community-dwelling population.

**Methods:**

The current analysis involved 898 individuals with carotid-femoral pulse wave velocity (cfPWV) ≤12 m/s. Central arterial stiffness was assessed by standard cfPWV at baseline and follow-up.

**Results:**

Incidence of cfPWV >12 m/s was 21.3% (102 participants). Participants without T2D had an increase of cfPWV with a median of 0.6 m/s, whereas participants with T2D had an increase of cfPWV with a median of 1.2 m/s (p = 0.007). T2D had an independent effect on increased cfPWV in multivariate Logistic regression models (p < 0.05 for all). Elevated levels of both FBG and PBG determined the independent effect on increased cfPWV in multivariate linear regression models (p < 0.05 for all).

**Conclusions:**

Type 2 diabetes had an independent effect on the development of central arterial stiffness in Chinese community-dwelling population. Both FBG and PBG should be responsible for the development of central arterial stiffness and treated as the targets of glycemic control.

## Background

Type 2 diabetes (T2D) places the individuals at high risk for developing the cardiovascular disease (CVD) [1, 2]. Since T2D and CVD are very common in China, the underlying mechanisms between them have been recognized as a significant public health issue and not been fully elucidated in public health field [3]. Central arterial stiffness may be a significant pathway linking T2D to CVD risk [4]. Central arterial stiffness has been considered as an useful marker of atherosclerosis and a significant determinant of CVD risk [5–7]. Several epidemiological studies have reported that central arterial stiffness predicts the mortality and morbidity of CVD [8–12]. Among the noninvasive methods available to assess the central arterial stiffness, carotid-femoral pulse wave velocity (cfPWV) has been accepted as the gold standard due to its reliability and validity [13–16].

Cardiovascular disease constitutes a major challenge for the health of Chinese community-dwelling population, it is essential to delay the development of atherosclerosis in this population. In previous studies, central arterial stiffness increases after glucose ingestion, and increased blood glucose after glucose ingestion is an independent risk factor for cardiovascular risk [17, 18]. However, there is a lack of long-term prospective studies analyzing the effect of T2D on cfPWV, especially in Chinese community-dwelling population [19]. Moreover, roles of fasting blood glucose (FBG) and postprandial blood glucose (PBG) in this effect are controversial, with some studies emphasizing PBG rather than FBG and other studies concerning FBG but not PBG [20–22]. Recent study has suggested that increased cardiovascular risk due to postprandial hyperglycemia might be associated with arterial dysfunction and stiffening, and it is important to examine the effect of FBG and PBG on central arterial stiffness [23]. Purpose of the current analysis was to investigate the effect of T2D on cfPWV during the 5 years of follow-up, and explore whether both FBG and PBG were determinants of this effect in Chinese community-dwelling population.

## Methods

### Study population

The current analysis recruited 1680 individuals of Han origin aged 18 years or older who voluntarily participated in a large medical check-up program at the health service centers in Beijing from May 2007 to July 2009. In the first stage of sampling, three districts (Fengtai, Shijingshan and Daxing) were selected from 18 districts in Beijing. In the second stage of sampling, four communities were selected from these districts. In the third stage of sampling, participants were selected from these communities. The follow-up visit was performed from February 2013 to September 2013. There were 181 participants lost, and follow-up of 5 years was completed for 1499 participants. Exclusion criteria were cfPWV >12 m/s (451 participants), missing values for variables (127 participants) and death (52 participants). There were 898 participants available for final analysis.

### Physical examination

Physical examination was performed by trained physicians. Weight was measured with a digital scale, and height was measured with a wall-mounted measuring tape, with light clothes and no shoes. Body mass index (BMI) was calculated as weight (kg) divided by squared height (m^2^). Blood pressure was measured in the sedentary position with a standard mercury sphygmomanometer (Yuwell Medical Equipment & Supply Co. Ltd. Jiangsu, China) after a 5-min rest. Adequate-sized cuff, based on arm circumference, was applied around the right arm comfortably placed at heart level [24]. The first and fifth phases of Korotkoff sounds were used as systolic blood pressure (SBP) and diastolic blood pressure (DBP), respectively. SBP and DBP were calculated as the mean of two readings with an interval of at least 1 min. Hypertension was diagnosed with mean SBP ≥140 mmHg, mean DBP ≥90 mmHg, and/or use of anti-hypertensive drugs.

### Laboratory analysis

Blood samples were drawn from participants between 8 and 10 a.m. after overnight fasting for at least 12 h, and submitted to our central laboratory. Blood triglycerides (TG), highdensity lipoprotein cholesterol (HDL-c), low-density lipoprotein cholesterol (LDL-c) and FBG were analyzed by qualified technicians blinded to clinical data using enzymatic assays (Hoffman-La Roche Ltd., Basel, Switzerland) on a fully automatic biochemical analyzer (Cobas 6000; Hoffman-La Roche Ltd.). Standard oral glucose tolerance test was performed 2 h after consumption of 75 g glucose to determine the PBG. T2D was diagnosed with FBG ≥7.0 mmol/L, PBG ≥11.1 mmol/L, and/or use of insulin or oral anti-diabetic drugs [25]. High TG was defined as TG ≥2.26 mmol/L (200 mg/dL), low HDL-c as HDL-c <1.04 mmol/L (40 mg/dL), and high LDL-c as LDL-c ≥4.14 mmol/L (160 mg/dL), and/or use of anti-dyslipidemic drugs [26, 27].

### Central arterial stiffness assessment

Central arterial stiffness was assessed by automated measurement of cfPWV using Complior Colson device (Créatech, Besançon, France) at baseline and follow-up; the technical characteristics of this device have been described [28]. cfPWV along the artery was measured with two strain-gauge transducers (TY-306 Fukuda pressure-sensitive transducer; Fukuda Denshi Co. Tokyo, Japan) fixed transcutaneously over the course of arteries separated by a known distance; the carotid and femoral arteries (all on the right side) were used. After pulse waveform of sufficient quality was recorded, the digitization process was initiated by the operator, and automatic calculation of the time delay between two upstrokes was started. Measurements were repeated over ten different cardiac cycles, and mean value was used for the final analysis. cfPWV was calculated from the measurement of the pulse transit time and the distance traveled by the pulse between two recording sites (measured on the surface of the body in meters), according to the following formula: cfPWV (m/s) = distance (m)/transit time (s).

### Statistical analysis

Continuous variables with normal distribution were presented as mean and standard deviation, whereas continuous variables with skewed distribution were presented as median and interquartile range. Categorical variables were presented as number and percentage. Based on the the diagnostic standard of an increase in cfPWV recommended by the international guideline, groups were determined according to cfPWV >12 or ≤12 m/s at follow-up, and continuous variables were compared between groups using Student’s t test (normal distribution) and Mann–Whitney U test (skewed distribution) [29]. Chi squared test was performed for comparative analysis of categorical variables. Wilcoxon signed-rank test was used to evaluate the change of cfPWV at baseline and follow-up. Pearson and Spearman correlations were used to evaluate the bivariate correlations. Logistic regression analysis was used to evaluate the effect of T2D on cfPWV >12 or ≤12 m/s at follow-up with adjustment for confounding factors (model 1: no adjustment; model 2: adjusted for age ≥60 years and gender; and model 3: adjusted for age ≥60 years, gender, smoker, drinker, obesity, coronary artery disease (CAD), hypertension, high TG, low HDL-c, high LDL-c, anti-hypertensive drugs, anti-dyslipidemic drugs and anti-diabetic drugs). Meanwhile, change of cfPWV was transformed to meet the multinormality assumption, and then linear regression analysis was used to evaluate whether both FBG and PBG were determinants of this effect on change of cfPWV with adjustment for confounding factors (model 1: no adjustment; model 2: adjusted for age and gender; and model 3: adjusted for age, gender, smoker, drinker, body mass index, SBP, DBP, TG, HDL-c, LDL-c, anti-hypertensive drugs, anti-dyslipidemic drugs and anti-diabetic drugs). Statistical analysis was performed using statistical package for the social science (SPSS) software (version 17.0; SPSS Inc. Chicago, IL, USA), with the level of significance set at two sided p < 0.05.

## Results

The current analysis had a median age of 53 (range 23–96) years, with 401 males and 497 females. Prevalence of T2D was 11.4% (102 participants) at baseline. Median cfPWV were 9.8 (8.8–10.7) and 10.3 (9.2–11.7) m/s at baseline and follow-up, respectively. Change of cfPWV was 0.7 m/s (p < 0.001 for change) and incidence of cfPWV >12 m/s was 21.3% (191 participants) at follow-up. Participants without T2D had an increase of cfPWV with a median of 0.6 m/s, whereas participants with T2D had an increase of cfPWV with a median of 1.2 m/s (p = 0.007). As presented in Table 1, age, SBP, FBG and PBG levels were significantly higher in participants with cfPWV >12 m/s than those with cfPWV ≤12 m/s (p < 0.05 for all). Percentages of age ≥60, males, CAD, hypertension and T2D in participants with cfPWV >12 m/s were significantly higher than those with cfPWV ≤12 m/s (p < 0.05 for all).

**Table 1:** Characteristics of study population grouped by cfPWV ≤12 or >12 m/s at follow-up

Characteristics	Total (n = 898)	CfPWV >12 m/s (n = 707)	CfPWV ≤ 12 m/s (n = 191)	p value^a^	p value^b^
Age (years)	53 (48–61)	52 (46–59)	63 (54–71)	<0.001	<0.001
Age ≥60 (%)	285 (31.7)	168 (23.8)	117 (61.3)	<0.001	<0.001
Males (%)	401 (44.7)	302 (42.7)	99 (51.8)	0.025	0.563
Smoker (%)	210 (23.4)	161 (22.8)	49 (25.7)	0.404	0.188
Drinker (%)	190 (21.2)	155 (21.9)	35 (18.3)	0.280	0.001
BMI (kg/m^2^)	25.23 (23.42–27.55)	25.24 (23.44–27.40)	25.14 (23.18–27.73)	0.782	0.363
Obesity (%)	186 (20.7)	143 (20.2)	43 (22.5)	0.489	0.521
CAD (%)	77 (8.6)	45 (6.4)	32 (16.8)	<0.001	0.010
SBP (mmHg)	123 (112–136)	122 (111–132)	131 (120–145)	<0.001	0.350
DBP (mmHg)	77 (70–83)	77 (70–83)	79 (70–86)	0.217	0.039
Hypertension (%)	323 (36.0)	213 (30.1)	110 (57.6)	<0.001	0.019
TG (mmol/L)	1.42 (1.02–2.08)	1.40 (1.01–2.01)	1.47 (1.06–2.31)	0.096	0.460
High TG (%)	194 (21.6)	144 (20.4)	50 (26.2)	0.083	0.017
HDL-c (mmol/L)	1.37 (1.15–1.61)	1.37 (1.16–1.61)	1.37 (1.13–1.62)	0.791	0.998
Low HDL-c (%)	124 (13.8)	95 (13.4)	29 (15.2)	0.535	0.650
LDL-c (mmol/L)	2.85 (2.39–3.23)	2.84 (2.39–3.21)	2.91 (2.44–3.33)	0.227	0.821
High LDL-c (%)	38 (4.2)	28 (4.0)	10 (5.2)	0.437	0.939
FBG (mmol/L)	4.96 (4.60–5.39)	4.92 (4.59–5.31)	5.11 (4.64–5.65)	0.001	0.014
PBG (mmol/L)	6.09 (5.12–7.70)	5.93 (5.06–7.29)	6.82 (5.52–8.54)	<0.001	0.008
T2D (%)	102 (11.4)	66 (9.3)	36 (18.8)	<0.001	0.008
Anti-hypertensive drugs (%)	182 (20.3)	114 (16.1)	68 (35.6)	<0.001	0.007
Anti-dyslipidemic drugs (%)	40 (4.5)	31 (4.4)	9 (4.7)	0.846	0.953
Anti-diabetic drugs (%)	19 (2.1)	13 (1.8)	6 (3.1)	0.408	0.773

*cfPWV* carotid femoral pulse wave velocity, *BMI* body mass index, *SBP* systolic blood pressure, *DBP* diastolic blood pressure, *TG*: triglyceride, *HDL-c* high density lipoprotein cholesterol, *LDL-c* low density lipoprotein cholesterol, *FBG* fasting blood glucose, *PBG* postprandial blood glucose, *T2D* type 2 diabetes

^a^Simple comparison between groups determined by cfPWV ≤12 or >12 m/s at follow-up

^b^Bivariate correlations of all variables with change of cfPWV

Change of cfPWV had a significant correlation not only with levels of age, DBP, FBG and PBG, but also with percentages of age ≥60, CAD, hypertension, high TG and T2D (p < 0.05 for all; Table 1). T2D had an independent effect on increased cfPWV after adjustment in all three models (p < 0.05 for all; Table 2). Elevated levels of FBG determined the independent effect on increased cfPWV in all three models (p < 0.05 for all; Table 3). When FBG was replaced by PBG, elevated levels of PBG also determined the independent effect on increased cfPWV in all three models (p < 0.05 for all; Table 3).

**Table 2:** Logistic regression models to analyze the effect of T2D on cfPWV ≤12 or >12 m/s at follow-up

Characteristics	HR	95CI	p value
First model	2.256	1.449–3.511	<0.001
Second model	1.973	1.228–3.171	0.005
Third model	2.207	1.285–3.789	0.004

*First model* no adjustment, *Second model* adjusted for age ≥60 years and gender, *Third model* adjusted for age ≥60 years, gender, smoker, drinker, obesity, coronary artery disease, hypertension, high triglyceride, low high density lipoprotein cholesterol, high low density lipoprotein cholesterol, anti-hypertensive drugs, anti-dyslipidemic drugs and anti-diabetic drugs, *T2D* type 2 diabetes, *cfPWV* carotid femoral pulse wave velocity, *HR* hazard ratio, *CI* confidence interval

**Table 3:** Linear regression models to analyze the effect of FBG and PBG on change of cfPWV

Characteristics	FBG			PBG		
	Standardized β	t	p value	Standardized β	t	p value
First model	0.104	3.144	0.002	0.111	3.355	0.001
Second model	0.108	3.361	0.001	0.080	2.445	0.015
Third model	0.117	3.455	0.001	0.087	2.531	0.012

*First model* no adjustment, *Second model* adjusted for age and gender, *Third model* adjusted for age, gender, smoker, drinker, body mass index, systolic blood pressure, diastolic blood pressure, triglyceride, high density lipoprotein cholesterol, low density lipoprotein cholesterol, anti-hypertensive drugs, anti-dyslipidemic drugs and anti-diabetic drugs, *FBG* fasting blood glucose, *PBG* postprandial blood glucose, *cfPWV* carotid femoral pulse wave velocity

## Discussion

T2D has been proved as a predictor for CVD in previous studies [1, 2]. An individual with T2D is generally considered to have a similar CVD risk as that of an individual without T2D but with myocardial infarction [30]. Central arterial stiffness may be a significant pathway linking T2D to CVD risk. The technical reliability and validity of cfPWV make it feasible for quantifying the central arterial stiffness in a large population. In order to determine the effect of T2D on the development of atherosclerosis in Chinese community-dwelling population, the current analysis investigated the effect of T2D on cfPWV, and explored the roles of FBG and PBG in this effect during the 5 years of follow-up.

The current analysis realized that participants with T2D had significantly higher increase of cfPWV compared with participants without T2D. Previous study has reported that an increase of cfPWV by 1 m/s leads to a 15% higher risk of CVD death, and thus change of cfPWV has significant clinical consequences [11]. Moreover, the current analysis suggested that T2D played an independent effect on central arterial stiffness even after full adjustment, and provided the evidence that central arterial stiffness was a significant and detectable mechanism between T2D and CVD. Corroborating our findings, recent studies have also found the relationship between T2D and cfPWV in other populations [20, 31]. Central arterial stiffness increases after glucose ingestion, and increased blood glucose after glucose ingestion is an independent risk factor for cardiovascular risk [17, 18]. The following pathways appear to be involved in mediating between T2D and cfPWV: firstly, T2D induces the production of reactive oxygen species through activating the oxidative stress [32, 33]; secondly, T2D reduces the nitric oxide of endothelial cells and attenuates the sensitivity of smooth muscle cells to nitric oxide [34]; thirdly, T2D stimulates the release of proinflammatory cytokines and causes the chronic inflammation [33, 35]; fourthly, T2D changes the structure of elastin and collagen in the arterial wall through raised sympathetic activity [36, 37]; finally, elevated levels of advanced glycation end-product alter the significant matrix of molecules in the arterial wall, and result in the pathological changes of arterial wall [38–40].

There has been controversial results about the roles of FBG and PBG in the effect of T2D on central arterial stiffness. Some studies have found that individuals with elevated levels of PBG, but not FBG, are significantly associated with central arterial stiffness [19]. However, other studies have reported that elevated levels of FBG rather than PBG are significant risk factor for central arterial stiffness and CVD [20, 21]. Recent study has suggested that increased cardiovascular risk due to postprandial hyperglycemia might be associated with arterial dysfunction and stiffening, and it is important to examine the effect of FBG and PBG on central arterial stiffness [23]. The current analysis confirmed that both FBG and PBG had an independent effect on central arterial stiffness in Chinese community-dwelling population. Fasting hyperglycemia affects vascular function through a series of different mechanisms, and inflammation plays a vital role in its association with vascular stiffness [34]. The mechanism by which postprandial hyperglycemia impairs vascular function is incompletely understood, but one major contributor is decreased bioavailability of nitric oxide caused by oxidative stress [41]. Since both FBG and PBG were independent determinants of central arterial stiffness, clinical doctors should pay attention to both FBG and PBG as the targets of glycemic control, and avoid ignoring either one of FBG and PBG in the development of central arterial stiffness and CVD.

## Conclusions

This 5-year prospective analysis demonstrated that T2D had an independent effect on the development of central arterial stiffness in Chinese community-dwelling population. Moreover, both FBG and PBG should be responsible for the development of central arterial stiffness and treated as the targets of glycemic control.

## Abbreviations

T2D: type 2 diabetes
CVD: cardiovascular disease
cfPWV: carotid-femoral pulse wave velocity
FBG: fasting blood glucose
PBG: ostprandial blood glucose
SBP: systolic blood pressure
DBP: diastolic blood pressure
TG: triglycerides
HDL-c: highdensity lipoprotein cholesterol
LDL-c: low-density lipoprotein cholesterol
CAD: coronary artery disease
